# Giant cell arteritis with vertebral artery involvement—baseline characteristics and follow-up of a monocentric patient cohort

**DOI:** 10.3389/fneur.2023.1188073

**Published:** 2023-06-26

**Authors:** Mona Klara Ros Prünte, Anne Naumann, Monika Christ, Markus Naumann, Antonios Bayas

**Affiliations:** ^1^Department of Neurology and Clinical Neurophysiology, Faculty of Medicine, University of Augsburg, Augsburg, Germany; ^2^Department of Neurology, University of Erlangen-Nuremberg, Erlangen, Germany

**Keywords:** giant cell arteritis, vertebral artery, immunotherapy, tocilizumab, imaging

## Abstract

Vertebral artery (VA) involvement in giant cell arteritis (GCA) has rarely been reported. We aimed to evaluate the prevalence, patients’ characteristics, and immunotherapies used in patients with GCA and VA involvement at diagnosis and 1 year follow-up, retrospectively including patients being diagnosed between January 2011 and March 2021 in our department. Clinical features, laboratory data, VA imaging, immunotherapy, and 1 year follow-up data were analyzed. Baseline characteristics were compared to GCA patients without VA involvement. Among all 77 cases with GCA, 29 patients (37.7%) had VA involvement, as diagnosed by imaging and/or clinical signs and symptoms. Gender distribution and erythrocyte sedimentation rate (ESR) were significantly different in the groups with and without VA involvement, with more women being affected (38/48 patients, 79.2%) and a significantly higher median ESR in patients without VA involvement (62 vs. 46 mm/h; *p* = 0.012). MRI and/or CT showed vertebrobasilar stroke at GCA diagnosis in 11 cases. 67/77 patients (87.0%) received high-dose intravenous glucocorticosteroids (GCs) at diagnosis, followed by oral tapering. Six patients were treated with methotrexate (MTX), one with rituximab, and five with tocilizumab (TCZ). 2/5 TCZ patients achieved clinical remission after 1 year, vertebrobasilar stroke within the first year occurred in 2/5 patients. Diagnosis of VA involvement might be underrecognized in GCA patients. VA imaging should be performed in elderly patients with vertebrobasilar stroke presenting with GCA symptoms, not to miss GCA as the etiology of stroke. Efficacy of immunotherapies in GCA with VA affection and long-term outcomes need to be investigated further.

## Introduction

Giant cell arteritis (GCA) is an immune-mediated disease primarily affecting medium and large-sized extracranial vessels, commonly the branches of the external carotid artery. Intracranial vessels are usually spared (1–3). Vertebral artery (VA) involvement in GCA is rare compared to the typical affection of the superficial cranial vessels, and only few cases in this patient group have been reported in the literature (2, 4–13). VA involvement resulting from GCA may lead to progressive brainstem- and/or cerebellar syndromes and is associated with increased mortality rates (5, 14).

The prevalence of stroke in GCA ranges from 1.5% to 11% (7, 15–18). In contrast to the distribution of strokes in the general population, ischemic events in GCA patients primarily affect the vertebrobasilar territory (5, 16, 17, 19). Larger cohort studies of patients with VA involvement in GCA and data on the long-term course do not exist to the best of our knowledge.

Although glucocorticosteroids (GCs) remain the mainstay of treatment in GCA, long-term treatment is associated with side effects (20), and up to 70% of patients with GCA experience relapses (21). Steroid-sparing and recurrence-lowering therapeutics like methotrexate (MTX) (22) and tocilizumab (TCZ) (23) can be used in situations where GCs-related side effects have ensued or are anticipated. TCZ, a human monoclonal antibody directed against the interleukin-6 receptor, has been approved for patients with GCA (24). Its efficacy has been demonstrated in phase 2 (23), phase 3 studies (25), and case series (26). However, in the literature only sparse data exist on the efficacy and safety of TCZ in GCA patients with VA involvement (23, 25, 27, 28).

The aim of this study was to describe characteristics of a cohort of GCA patients with VA involvement focusing on clinical, laboratory and imaging data as well as treatments used.

## Patients and methods

### Study characterization

**Figure 1:**
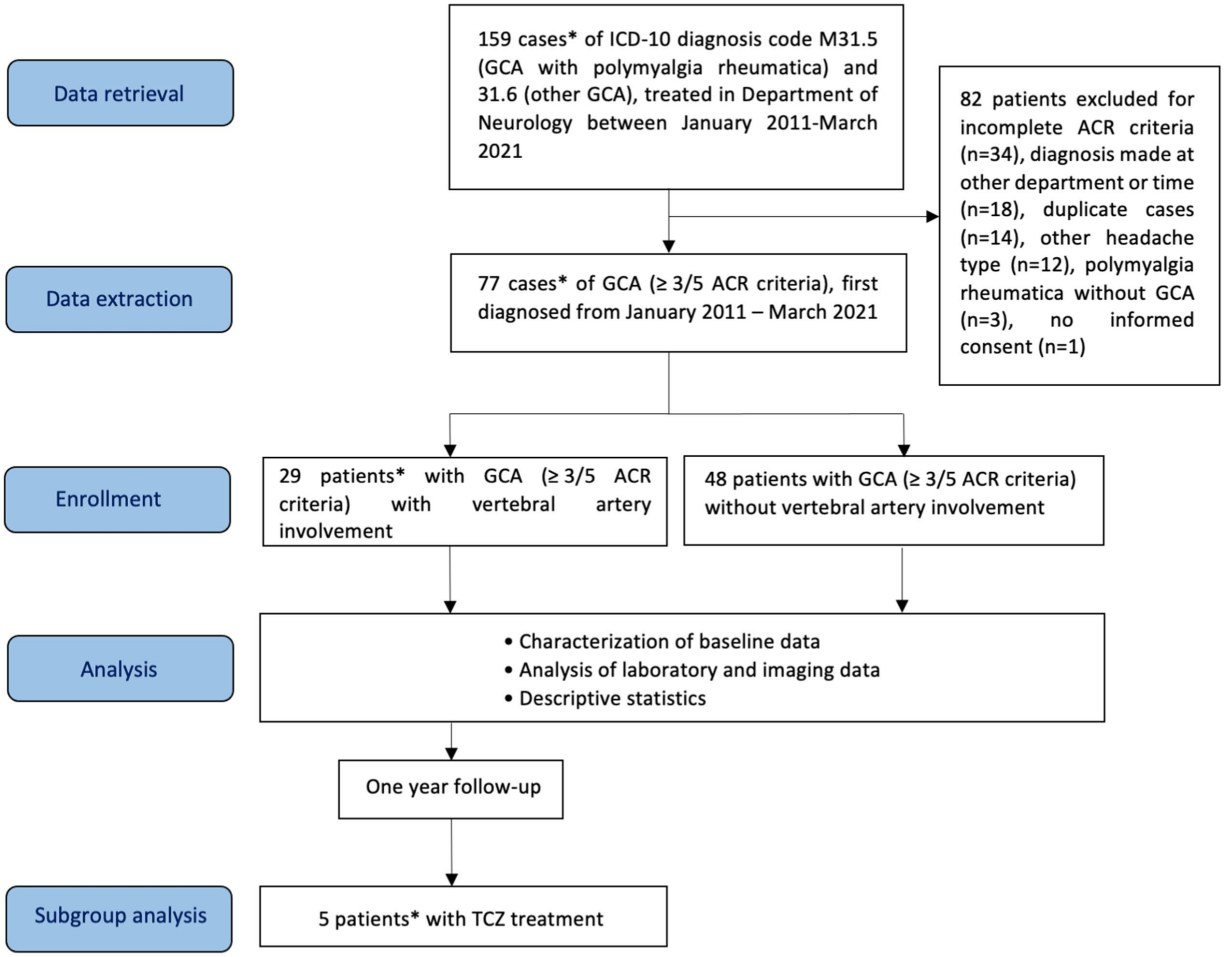
Flow diagram of patients included in the study, analysis and subgroup analysis. GCA, giant cell arteritis; ACR, American College of Rheumatology; TCZ, tocilizumab. *One patient was first diagnosed with vertebrobasilar ischemia and VA involvement in GCA in our department, the diagnosis of GCA was made 3.3 years earlier in the Department of Internal Medicine of the Hospital of Augsburg.

For this monocentric, mainly retrospective study, a total of 159 cases of GCA (ICD-10 (International Statistical Classification of Diseases and Related Health Problems) codes M31.5, GCA with polymyalgia rheumatica, and M31.6, other GCA), diagnosed in our department between January 2011 to March 2021, were identified retrospectively from the hospital’s electronic clinical information system (Figure 1). Diagnosis of GCA was based on the presence of at least 3 out of the following 5 criteria of the American College of Rheumatology (ACR): age ≥ 50 years at disease onset, new-onset headache, abnormal temporal arteries (tenderness, decreased pulsation), erythrocyte sedimentation rate (ESR) > 50 mm/h, and histology of temporal artery biopsy (vasculitis with predominantly mononuclear cell infiltration or granulomatous inflammation, usually with multinucleated giant cells) (29). Of note, the ACR classification criteria for GCA have been revised in 2022 (30).

### Data collection

Demographic data, concomitant medication, and cardiovascular risk factors, symptoms and clinical features, laboratory [c-reactive protein (CRP), ESR], histological and imaging data, and data regarding immunotherapy were collected retrospectively from electronic patient files. In 3/5 TCZ-treated patients, follow-up data were collected retro- and prospectively. Questionnaires collecting additional information on the disease course (symptoms, diagnostic findings, occurrence of stroke, and immunotherapy) within the first 2 years after diagnosis of GCA were sent out to patients. Patients who needed assistance in completing the questionnaire were contacted by phone or visited in person. In case of missing information, treating physicians outside our department were contacted. Data were collected for all patients at the time of first diagnosis of GCA, and for patients with VA involvement additionally at 3, 6, and 12 months thereafter. TCZ is the only drug specifically licensed for GCA, so one focus of our study were TCZ treated patients.

### Case definition

**Figure 2:**
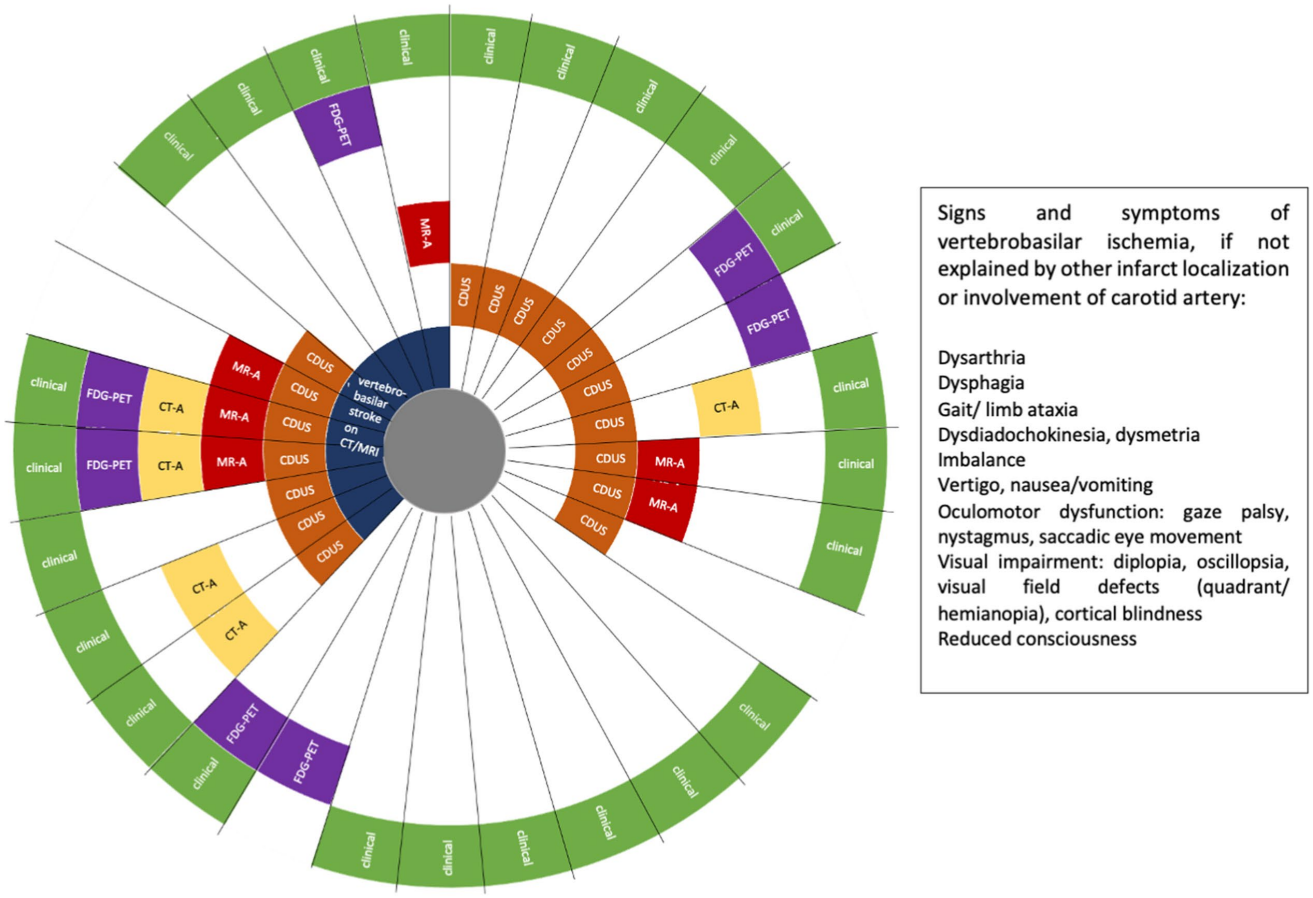
Patients with giant cell arteritis (GCA) and vertebral artery (VA) involvement (*n* = 29). FDG-PET, 18F-fluorodeoxyglucose positron emission tomography; CT-A, computed tomography angiography; MR-A, magnetic resonance angiography; CDUS, colour Doppler ultrasound. Each ray reflects one patient.

In patients fulfilling GCA diagnosis criteria, VA involvement was defined as evidence of VA stenosis or occlusion on CDUS, magnet resonance angiography (MR-A) and/or computed tomography angiography (CT-A), 18F-fluorodeoxyglucose (FDG) uptake of the VA, and/or indirectly by imaging evidence of vertebrobasilar ischemia on MRI or CT (Figure 2). A single imaging modality showing VA involvement was defined sufficient for establishing the diagnosis of GCA with VA involvement. In addition, the following findings were suggestive for vasculitis of the VA but were not defined as inclusion criteria: concentric hypoechoic wall thickening (“halo sign”) of the VA on CDUS, and mural edema and contrast enhancement of the VA on MR-A. If VA involvement was not detected by imaging, symptoms and focal neurologic signs clearly indicating involvement of the VA territory were also sufficient to be included in the GCA + VA group (Figure 2).

### Ethics statement

The Ethics Committee of the University of Erlangen-Nuremberg provided approval. The database of patients with VA involvement was pseudonymized, and informed consent was obtained to use baseline and follow-up data. For patients who had already died the next of family members was contacted to give his/her approval after confirming that the use of data was presumed to be in the patient’s best interest. Data of patients without VA involvement were collected for group comparisons. Their data were anonymized and compiled into aggregate data, so no informed consent was necessary according to the Ethics Committee.

### Statistical analysis

Microsoft Excel 2016 and IBM SPSS Statistics (version 24) were used for statistical calculations. All continuous data are presented as median and interquartile ranges (IQR). All categorial variables are expressed in absolute values and percentages. Median observational time was calculated using the date of initial diagnosis of GCA and the date of the last available diagnostic investigation (laboratory or imaging data) within the first year after diagnosis. Mann–Whitney *U*-test was performed to compare continuous variables between GCA patients with and without VA involvement. The distribution of categorial data was analyzed by Chi-square test. *p-*values <0.05 were considered statistically significant.

## Results

### Demographic data

Figure 1 shows the flow diagram for study procedures. A total of 29 patients with GCA and VA involvement (GCA + VA) and 48 patients without VA involvement (GCA-VA) were identified. One patient in the GCA + VA group (case 5, see Supplementary material) was diagnosed in the Department of Internal Medicine of our hospital, and presented with vertebrobasilar ischemia 3.3 years later in our department, where VA involvement was diagnosed. Although this patient does not fulfil all inclusion criteria (missing: being diagnosed at our department), we included him to increase the patient number in this rare disorder. Patients’ baseline data are shown in Table 1. Patients with inflammatory VA involvement were categorized according to treatment (TCZ- vs. non TCZ-treatment) and compared with patients without VA involvement. Patients with and without VA involvement showed similar distribution in most demographic items and cardiovascular risk factors. Gender distribution was the only significant difference between the groups GCA + VA and GCA-VA (*χ*^2^ = 4.968, df = 1, *p* = 0.026). Regarding the 3:1 female-to-male ratio known for GCA (31), the gender distribution in the group GCA + VA did not differ significantly in a statistical analysis using the chi-square goodness of fit test (*χ*^2^ = 1.728, df = 1.000, *p* = 0.189), whereas in the group GCA-VA there was a significantly higher proportion of women (*χ*^2^ = 3.375, df = 1, *p* = 0.066; corresponding to a female-to-male ratio of 3.8:1).

**Table 1:** Characteristics of patients with and without vertebral artery (VA) involvement.

		All GCA patients with VA involvement	TCZ treated patients	Patients without TCZ	Patients without VA involvement	*p* values comparing patients with and without VA involvement
∑		29 (100%)	5 (17.2%)	24 (82.8%)	48 (100%)	–
Biometric characteristics						
Age (years)**		75.1 (12)	71.5 (7.9)	75.8 (13.9)	76.5 (11)	0.15
Female/male		**16 (55.2%)/13 (44.8%)**	0 (0%)/5 (100%)	16 (66.7%)/8 (33.3%)	**38 (79.2%)/10 (20.8%)**	**0.026**^b^**; *χ***^2^ **= 4.968**
Observational time (d)**		323 (313.5)	381 (710)	248 (349.8)	–^a^	–
Death within 1^st^ year	Overall	3 (10.3%)	1 (20%)	2 (8.3%)	–^a^	–
	Due to GCA	2 (6.9%)	1 (20%)	1 (4.2%)	–^a^	–
CV risk factors						
History of smoking		5 (17.2%); [13/29 n/a]	2 (40%); [1 n/a]	3 (12.5%); [12 n/a]	5 (10.4%); [38 n/a]	0.388
Arterial hypertension		22 (75.9%)	3 (60%)	19 (79.2%)	28 (58.3%)	0.118
Diabetes mellitus		5 (17.2%)	1 (20%)	4 (16.7%)	9 (18.8%)	0.868
Hypercholesterolemia		10 (34.5%)	2 (40%)	8 (33.3%)	15 (31.3%)	0.769
Malignoma		3 (10.3%)	1 (20%)	2 (8.3%)	2 (4.2%)	0.286
Laboratory data at GCA diagnosis						
CRP (mg/dL)**		6.4 (5.8)	4.8 (5.3)	7.0 (6.4)	6.4 (12.2)	0.484
ESR (mm/h)**		**46 (39)**	41 (31.5)	48 (41)	**62 (26.8)**	**0.012** ^b^
Temporal artery biopsy						
Under GCs treatment	IV GCs	10 (34.5%)	2 (40%)	8 (33.3%)	11 (22.9%)	0.27
	Oral GCs	13 (44.8%)	3 (60%)	10 (41.7%)	23 (47.9%)	0.792
Without GCS treatment		1 (3.4%)	0 (0%)	1 (4.2%)	0 (0%)	0.195
No biopsy		5 (17.2%)	0 (0%)	5 (20.8%)	14 (29.2%)	0.24
Inflammatory infiltrate and/or giant cells		18 (75%)*	3 (60%)*	15 (78.9%)*	25 (73.5%)*	0.393
Diagnostic imaging at GCA diagnosis						
CDUS	Performed	28 (96.6%)	4 (80%)	24 (100%)	47 (97.9%)	0.715
	VA stenosis	17 (60.7)*	3 (75%)*	14 (58.3%)*	0 (0%)*	–
MR-A	Performed	16 (55.2%)	2 (40%)	14 (58.3%)	7 (14.6%)	**0.00** ^b^
	VA stenosis	7 (43.8%)*	2 (100%)*	5 (35.7%)*	0 (0%)*	–
CT-A	Performed	16 (55.2%)	2 (40%)	14 (58.3%)	6 (12.5%)	**0.00** ^b^
	VA stenosis	5 (31.3%)*	2 (100%)*	3 (21.4%)*	0 (0%)*	–
FDG-PET	Performed	14 (48.3%)	5 (100%)	9 (37.5%)	4 (8.3%)	**0.00** ^b^
	VA enhancement	7 (50%)*	5 (100%)*	2 (22.2%)*	0 (0%)*	–
Cerebral ischemia at GCA diagnosis						
Vertebrobasilar transient ischemic attack		16 (55.2%)	2 (40%)	14 (58.3%)	0 (0%)	–
Stroke on CT/MRI	CT and/or MRI performed	26 (89.7%)	3 (60%)	23 (95.8%)	31 (64.6%)	**0.015** ^b^
	Anterior circulation stroke	0 (0%)*	0 (0%)*	0 (0%)*	1 (3.2%)*	0.434
	Vertebrobasilar stroke	11 (42.3%)*	3 (100%)*	8 (34.8%)*	0 (0%)*	–
Treatment at GCA diagnosis						
High-dose IV GCs		27 (93.1%)	5 (100%)	22 (91.7%)	40 (83.3%)	0.217
TCZ		4 (13.8%)	4 (80%)	0 (0%)	0 (0%)	**0.008** ^b^
MTX		5 (17.2%)	0 (0%)	5 (20.8%)	1 (2.1%)	**0.016** ^b^
RTX		1 (3.4%)	1 (20%)	0 (0%)	0 (0%)	0.195

* refers to group of patients for whom respective examination (biopsy or imaging) was performed. Continuous data (**) are represented as median (interquartile range), categorial data are represented as absolute values (%). Missing information is indicated in square brackets as not available [n/a]. GCA, giant cell arteritis; VA, vertebral artery; CV, cardiovascular; n/a, not available; CRP, c-reactive protein; ESR, erythrocyte sedimentation rate; GCs, glucocorticosteroids; IV, intravenous; CDUS, colour Doppler ultrasound; MR–A, magnetic resonance angiography; CT-A, computed tomography angiography; FDG-PET, 18F-fluorodeoxyglucose positron emission tomography; TCZ, tocilizumab; MTX, methotrexate; RTX, rituximab.

a Information on observational time and death not presented as only baseline data are displayed.

b Significant differences between group with and without VA involvement.The bold values highlight the significantly different values between the groups with and without VA involvement.

### Clinical assessment and treatment

In 6/29 patients (20.7%) of the GCA + VA group, imaging was not indicative for VA involvement at diagnosis, but those patients had symptoms and focal neurologic deficits that indicated ischemia in the vertebrobasilar territory (Figure 2). In total, neurological examination at first admission revealed focal neurologic deficits indicating vertebrobasilar involvement in 17/29 cases (58.6%). 14/29 patients (48.3%) in the GCA + VA group had VA stenosis demonstrated by at least one vascular imaging (CDUS, MR-A, and/or CT-A) at GCA diagnosis. Focusing on these patients, 6/14 patients (42.9%) had vertebrobasilar stroke on MRI and/or CT, 6/14 patients (42.9%) had a vertebrobasilar transient ischemic attack (TIA), and 2/14 patients (14.3%) had no symptoms related to an ischemic event. All 14 patients with VA stenosis were treated with high-dose intravenous (IV) methylprednisolone, 4/14 patients (28.6%) received additional MTX, 3/14 patients (21.4%) received TCZ. 2/14 patients (14.3%) had recurrent vertebrobasilar stroke within the first year of immunotherapy, one of them receiving oral prednisolone plus TCZ, one of them oral prednisolone plus MTX. In total, 27/29 patients (93.1%) with VA involvement and 40/48 patients (83.3%) without VA involvement were treated with high-dose IV GCs at GCA diagnosis, followed by oral tapering. The remaining patients received oral GCs. Six patients received MTX, one patient rituximab. 5/29 patients with VA involvement were treated with TCZ. Of note, one patient has been treated with rituximab for two years until he was switched to TCZ approximately 3.3 years after GCA diagnosis. One TCZ patient received TCZ only for one month due to elevated liver enzymes.

One year after GCA diagnosis, 3/29 patients of the GCA + VA group had died, with relation to GCA in two of them (recurrent strokes in the vertebrobasilar territory, resulting from occlusion or pre-occlusive stenosis of the vertebral arteries, respectively). 2/5 patients (40%) treated with TCZ died within (recurrent vertebrobasilar stroke) or shortly after (septic shock due to soft tissue infection of the leg) the first year of TCZ treatment. 2/5 patients (40%) were in clinical remission without stroke recurrence after 12 months of TCZ treatment. A more detailed description of the follow-up of TCZ treated patients is depicted and in the case descriptions in the supplement in Supplementary Figure S1.

### Laboratory and histological characterization

At GCA diagnosis, CRP (patients with and without VA affection: 6.4 mg/dL (median); reference <0.5 mg/dL) and ESR (patients with VA affection: 46 mm/h, patients without VA affection: 62 mm/h (median); reference <20 mm/h; *p* = 0.012) were elevated, as expected (Table 1). In 24/29 patients (82.8%) serologic vasculitis parameters (including antinuclear antibodies, extractable nuclear antigen antibodies, anti-neutrophil cytoplasmic antibodies, and antiphospholipid antibodies) and in 10/29 patients (34.5%) cerebrospinal fluid analysis, both analyzed in the context of initial differential diagnosis workup and not to confirm GCA, were unremarkable.

GCA was biopsy-proven in 18/24 patients (75%) with VA involvement who underwent temporal artery biopsy, and in 25/34 patients (73.5%) without VA involvement.

After 12 months of immunotherapy, in the group GCA + VA, median CRP was lower in the TCZ-group (median CRP 0.06 mg/dL) than in the non-TCZ group (median CRP 2.6 mg/dL), which can be explained by IL 6-receptor blockade affecting CRP and ESR. Regarding cholesterol and liver enzymes in TCZ patients, we found no relevant laboratory adverse reactions (hypercholesterolemia, elevation of liver transaminase levels) within the first year of treatment.

### Imaging for VA involvement

At the time of GCA diagnosis, 75/77 patients (97.4%) had VA imaging (28/29 patients in the GCA + VA group, one patient later during disease course, and 47/48 patients in the GCA-VA group). VA involvement was detected most often by CDUS (Table 1) with 17/28 patients (60.7%) revealing VA stenosis or occlusion of at least one VA. 14/17 patients (82.4%) with VA stenosis in CDUS had also hypoechogenic wall thickening (halo sign) of the VA. In 13/29 cases (44.8%), more than one imaging modality (CDUS, MR-A, CT-A, FDG-PET) showed VA involvement (Figure 2). FDG-PET was performed in 14/29 patients (48.3%) of the GCA + VA group, 9/14 patients (64.3%) showed FDG uptake of the aorta (aortitis) and its major branches. In the TCZ treated group, 4/5 patients had CDUS at the time of GCA diagnosis, showing VA affection in 3 of them. 2/3 patients showed decrease of VA stenosis during the first year of treatment (Supplementary Figure S1), the third patient died after 6 months of treatment. At TCZ treatment start, 4 patients had VA involvement as shown in FDG-PET. Two patients showed increase of VA FDG uptake after 6 months but decrease of FDG uptake after 12 months. One patient was negative in FDG-PET at TCZ initiation and after 12 months of treatment, respectively.

### Stroke in patients with and without VA involvement

Stroke at the time of GCA diagnosis was detected in 12/57 patients (20.1%) who had received brain imaging, and in 11/26 patients (42.3%) with VA involvement, respectively, in the latter group all in the vertebrobasilar territory. VA stenosis or occlusion (confirmed by CDUS, MR-A, and/or CT-A) and/or FDG uptake were detected in 9/11 patients (81.8%) at time of vertebrobasilar stroke. 16/29 patients (55.2%) presented with vertebrobasilar TIA. Two patients had no clinical signs of vertebrobasilar stroke or TIA, they were included based on VA stenosis in CDUS during initial routine GCA diagnostic workup. In one of 31 patients (3.2%) of the GCA-VA group who had received CT and/or MRI, a stroke was detected in the anterior circulation.

Within the first year after GCA diagnosis, recurrent stroke occurred in 2/5 TCZ treated patients (20%) and in 1/24 non-TCZ treated patients (4.2%).

## Discussion

There are only sparse data on characteristics of patients diagnosed with GCA and VA involvement in the literature. Here, we report characteristics of GCA patients with and without VA involvement and one year follow-up data of patients with VA involvement contributing important information on this disorder.

Considering that the prevalence of large artery involvement in GCA including the VA has been reported in the literature to be up to 15% (32), the prevalence of VA involvement among all GCA patients (29/77 patients; 37.7%) in our study is much higher which has not been reported hitherto. However, since this was a single center study exclusively including patients from a neurological department our findings may be explained by a selection bias for GCA patients with neurologic manifestations.

Published first in November 2022, the ACR classification criteria for GCA have been revised very recently (30). Since our study was initiated already in 2021, the 1990 ACR-criteria have been used.

Our results show that at diagnosis, characteristics of patients with and without VA involvement were similar for nearly all parameters investigated apart from the gender distribution showing no significant male to female difference in the GCA + VA group.

Identifying VA involvement in GCA is important as it may result in ischemia in the vertebrobasilar territory, which is associated with high mortality (5). In our study, stroke at GCA diagnosis was detected in 12/57 patients (21.1%) by brain imaging, which is higher than published stroke rates of 2.4%–2.8% in the literature at the time of GCA diagnosis (between onset of GCA symptoms/signs and 1 month after beginning of GCs therapy) (33). Again, this may be explained by the fact that we only included patients treated at our department of neurology resulting in a selection bias. Another reason may be the high proportion of patients with VA involvement in our study (37.7%), most of whom were admitted for symptoms in the vertebrobasilar territory accompanied by typical GCA symptoms. In the group of patients without VA involvement, stroke at the time of GCA diagnosis was present in 2.1%, which is in line with data from the literature.

In the literature, 50%–75% of GCA-related strokes occur in the vertebrobasilar territory (16), whereas an even higher proportion of vertebrobasilar ischemic events (91.7%) was observed in our study indicating that the prevalence of GCA-related ischemic events in the vertebrobasilar territory might be higher than previously assumed. In a systematic literature review on GCA and ischemic stroke by Elhfnawy et al., of 136 stroke patients with concomitant GCA, 70% had multiple stenoses/occlusions in the vertebrobasilar territory, which was considered as a red flag for GCA among patients with vertebrobasilar territory stroke (34). In our study, 9/11 patients (81.8%) with vertebrobasilar stroke at the time of GCA diagnosis had imaging evidence of VA stenosis or occlusion, or VA FDG uptake. In a recent multicenter study, multiple stenoses in the vertebrobasilar arteries were identified among 9/12 patients (75%) with GCA-related vertebrobasilar stroke, which is similar to our findings (17). In another study, abnormalities of the VA in GCA patients with vertebrobasilar ischemia were reported in 69% on CDUS (bilateral occlusion, halo), 86% on CT-A/MR-A (inflammatory wall thickening, stenosis), and 50% on FDG-PET (hypermetabolism) (18).

One focus of our study was the follow-up of five patients treated with TCZ, the first drug specifically approved for the treatment of GCA. The beneficial effects of TCZ as a GCs-sparing treatment have been established in two randomized trials (23, 25). In our study, after 1 year of TCZ treatment, imaging findings were improved in 2/5 cases (decrease of VA stenoses in CDUS and/or of VA FDG uptake; Supplementary Figure S1). In contrast, FDG-PET activity persisted in two cases despite stable clinical findings at 6-month follow-up. This is not unusual as it has been reported previously that disease activity shown by imaging may differ from clinical assessment (35, 36). FDG-PET is a powerful tool for the diagnosis of GCA (37), but there are no consistent data regarding its relevance for the assessment of disease progression and prognosis. In our study, FDG-PET results did not correlate well with the clinical course, at least in the first months of TCZ treatment.

Two patients suffered from recurrent ischemia in the vertebrobasilar territory within the first year of therapy and died 6 and 14 months, respectively, after TCZ initiation. It is likely that TCZ contributed to the soft tissue infection and sepsis in case 5. It has been reported that the use of TCZ in GCA may account for the increased risk of severe infection (38), so a causal role cannot be excluded. TCZ treatment was well tolerated by 4 of 5 patients within 1 year of treatment. In case 3, assessment of efficacy as well as side effects is limited due to the short period of TCZ treatment of one month.

In our study, ongoing disease activity was observed in 2/4 patients during treatment with TCZ, clinically and/or by imaging (new ischemia, persistent vascular inflammation on imaging). Due to the low number of TCZ treated patients, no general conclusions can be drawn. However, based on the small number reported, TCZ did not prevent ischemic recurrences in some patients. This must be investigated further in larger studies. To our knowledge, there are no data confirming the efficacy and safety of long-term TCZ treatment in GCA patients with VA affection. In a recent case report of GCA with VA affection, progression of VA stenosis and recurrent stroke despite aggressive immunotherapy (high-dose GCs, cyclophosphamide, TCZ) was observed with a fatal outcome (7). Case reports with similar clinical course, i.e., recurrent vertebrobasilar ischemia in GCA with VA affection despite intensive immunotherapy with TCZ or cyclophosphamide, have been published (5, 13, 32). In patients with stroke due to progressive GCA who are non-responsive to immunotherapy or in patients with severe symptomatic stenosis based on an underlying inflammatory process, endovascular therapy (EVT) (e.g., angioplasty) may be considered (39). It should be noted, however, that a higher rate of restenosis is observed after EVT (40, 41). It remains to be determined whether VA involvement in GCA requires more intensive treatment than in GCA without VA involvement.

As a strength, our study substantially contributes to the literature as it characterizes a relative high number of GCA patients with VA affection compared with the existing literature (2, 4–12). However, there are some limitations. First, we only included patients diagnosed at our institution (with one exception), which results in a selection bias for patients with neurological manifestation. Second, due to the predominantly retrospective study design, follow-up data were missing in a relevant proportion of patients, especially in the non-TCZ group, impeding comparisons of the long-term course of patients with and without VA affection. Third, VA involvement by GCA shown by imaging was assessed by CDUS, MR-A, CT-A and /or FDG-PET. We analyzed imaging results most stringently to exclude other etiologies. However, we cannot exclude that other etiologies like atherosclerosis may have contributed to VA pathology in select cases. Regarding this, the generally higher prevalence of atherosclerosis in males may have had an impact on the fact that more males are affected in the GCA + VA group.

However, the findings of our study provide important information that should make healthcare professionals aware of a possible VA involvement in patients diagnosed with GCA, since prompt and aggressive treatment may prevent complications. Larger prospective studies would be necessary, especially concerning the long-term outcome and efficacy of immunotherapies. However, based on the rarity of the disease, this is difficult to achieve making studies like our work more important.

## Conclusion

Stroke in the vertebrobasilar territory presenting with typical GCA symptoms should raise the suspicion of GCA, especially if no other cause of stroke can be identified. Normal CRP or ESR does not rule out GCA. CDUS, possibly also MR-A, and FDG-PET in select cases should be performed.

Results from previous studies showing that the combination of TCZ and GCs is more effective for maintaining clinical remission in GCA than GCs monotherapy cannot be confirmed or disproved in our study due to the small number of cases.

## Data availability statement

The raw data supporting the conclusions of this article will be made available by the authors, without undue reservation.

## Author contributions

MP drafting the manuscript, acquisition of data, data interpretation, literature search, statistical analysis, visualization of data, and accessed and verified the underlying data. AN acquisition of data, data interpretation, literature search, visualization of data, and accessed and verified the underlying data. MC and MN data interpretation and revision of the manuscript for intellectual content, and accessed and verified the underlying data. AB drafting the manuscript, acquisition of data, data interpretation, literature search, and accessed and verified the underlying data. All authors contributed to the article and approved the submitted version.

## Conflict of interest

The authors declare that the research was conducted in the absence of any commercial or financial relationships that could be construed as a potential conflict of interest.

## Publisher’s note

All claims expressed in this article are solely those of the authors and do not necessarily represent those of their affiliated organizations, or those of the publisher, the editors and the reviewers. Any product that may be evaluated in this article, or claim that may be made by its manufacturer, is not guaranteed or endorsed by the publisher.

## Supplementary material

The Supplementary material for this article can be found online at: https://www.frontiersin.org/articles/10.3389/fneur.2023.1188073/full#supplementary-material

## Figures and Tables

**Table:** 

VA	vertebral artery/ arteries
GCA	giant cell arteritis
ACR	American College of Rheumatology
CDUS	colour Doppler ultrasound
MRI	magnetic resonance imaging
MR-A	magnetic resonance angiography
CT	computed tomography
FDG-PET	18F-fluorodeoxyglucose positron emission tomography
ICD	International Statistical Classification of Diseases and Related Health Problems
TIA	transient ischemic attack
IV	intravenous
GCs	glucocorticosteroids
MTX	methotrexate
TCZ	tocilizumab
ESR	erythrocyte sedimentation rate
CRP	c-reactive protein
EVT	endovascular therapy
