# Supplementary material for: Giant cell arteritis with vertebral artery involvement—baseline characteristics and follow-up of a monocentric patient cohort
Source: Front Neurol. 2023 Jun 26;14:1188073. doi: 10.3389/fneur.2023.1188073 (PMC10331602; doi:10.3389/fneur.2023.1188073)
Supplement: Supplementary file 1 [file Data_Sheet_1.PDF]

Supplement

Case descriptions

Case 1:

A 71-year-old man suffered from vertebrobasilar stroke (bilateral cerebellum and mesial temporal lobe on magnet resonance imaging (MRI)) with gait ataxia, dizziness, and Horner's syndrome. Computed tomography angiography (CT-A) revealed multisegmental medium- to high-grade stenoses of the left vertebral artery (VA) and occlusion of the right VA. Colour doppler ultrasound (CDUS) showed signs of giant cell arteritis (GCA) with a halo sign of the proximal superficial temporal artery on both sides and stenoses of both VA. 18F-fluorodeoxyglucose (FDG) positron emission tomography (PET) and black blood MRI angiography showed marked vasculitic changes of both VA. Laboratory investigation was normal except for elevated C-reactive protein (CRP; 7.87 mg/dl; reference <0.5 mg/dl) and mild elevation of erythrocyte sedimentation rate (ESR; 41 mm/h; reference <20 mm/h). Temporal artery biopsy confirmed the diagnosis of GCA with typical histopathologic findings (inflammatory infiltrate, numerous Langerhans giant cells). Intravenous (IV) high-dose methylprednisolone (MP), followed by oral prednisolone tapering, was initiated and dual antiplatelet therapy combining ASS and clopidogrel for peripheral artery disease was continued. Steroid-sparing therapy with tocilizumab (TCZ) was initiated and administered at a dose of 162 mg s.c. weekly. At 3-month follow-up, clinical examination revealed an unchanged cerebellar atactic syndrome. MRI, which was performed for suspected sonographic progression of the VA stenoses and frequent presyncopal episodes, showed no new cerebral ischemia but increasing stenosis of the left VA and new contrast enhancement of the basilar artery with segmental stenosis. CDUS was stable. Recurrent pre-syncopes and persistent dizziness were considered correlates of the vertebrobasilar stenoses. The patient was treated with high-dose MP again. Approximately 5 months after initiation of TCZ therapy, the patient presented comatose. Cranial CT showed new infarcts in the thalami, brainstem and cerebellum bilaterally, most likely resulting from bilateral posterior and basilar artery occlusion, and brain herniation based on generalized cerebral edema. The patient died almost 6 months after diagnosis of GCA.

Case 2:

A 76-year-old male presented with recurrent amaurosis fugax and transient diplopia. Cranial CT with angiography showed no ischemia but irregularities of both VA with bilateral distal occlusion, suggesting vasculitis. CDUS revealed hypoechoic wall thickening of both VA with hemodynamically significant stenosis of the right VA. The superficial temporal artery showed a halo sign, consistent with GCA. CRP (3.95 mg/dl) and ESR (61 mm/hour) were elevated. High-dose MP was administered, followed by oral prednisolone tapering. Temporal artery biopsy was performed under glucocorticosteroid (GCs) therapy and did not show typical histological signs of GCA. FDG-PET showed FDG-uptake of both VA. TCZ treatment was initiated in addition to GCs treatment. At 3-month follow-up, the patient had no symptoms and signs of GCA. CDUS showed new left VA occlusion and unchanged stenosis of the right VA. At 6-month follow-up, vascular status remained unchanged on CDUS, although FDG-PET showed mild progressive enhancement of the VA. Due to stable disease based on clinical and sonographic findings, prednisolone was further tapered and TCZ continued. 12 months after initiation of TCZ treatment, the patient remained asymptomatic and vascular imaging (CDUS, FDG-PET) of the VA showed improvement. Prednisolone treatment was discontinued.

Case 3:

A 71-year-old man suffered from transient ischemic attack in the vertebrobasilar territory with hemiparesis and dysarthria. There was no cerebral ischemia on CT. ASS and rosuvastatin for secondary prevention were administered. Routine CDUS showed hypoechoic wall thickening of the left superficial temporal artery and both VA without stenosis. CRP (8.15 mg/dl) and ESR (25 mm/h) were elevated. FDG-PET confirmed large vessel vasculitis with involvement of both VA. The patient was treated with high-dose MP, followed by oral prednisolone tapering. Temporal artery biopsy was performed under GCs therapy, histopathological features of GCA could not be detected. TCZ was initiated. At 3-month follow-up, the patient reported infrequent attacks of dizziness, clinical examination was unremarkable. TCZ was discontinued 29 days after initiation of therapy for elevated liver enzymes. Despite normalization of liver enzymes 4 months after start of TCZ, the patient refused continuing TCZ therapy

due to a newly diagnosed prostate carcinoma. At 6- and 12-months follow-up, clinical and sonographic findings were unremarkable. Prednisolone monotherapy was slowly reduced.

Case 4:

A 72-year-old male presented with recurrent episodes of dizziness, bitemporal headache, and transient monocular blurred vision with metamorphopsia. MRI revealed bilateral cerebellar infarcts. Irregularities of both VA with hypoechoic wall thickening of both VA and increased flow rates of the left VA, and a halo sign of both superficial temporal arteries on CDUS led to the diagnosis of GCA. FDG-PET confirmed vasculitis of the VA. ESR (31 mm/h) and CRP (1.41 mg/dl) were mildly elevated. Diagnosis of GCA was also confirmed histologically with typical findings of GCA (inflammatory infiltrate, giant cells). The patient received ASS and high-dose MP therapy, followed by oral prednisolone tapering. Furthermore, weekly TCZ (162 mg s.c.) was initiated. After 3 months, the patient was asymptomatic except for rare episodes of headache. Flow acceleration of the left VA remained unchanged. At 6-month follow-up, VA stenosis was no longer detectable by CDUS. FDG-PET showed ongoing active vasculitis with mildly progressive enhancement of the VA. The patient remained clinically stable. Cranial MRI showed no new ischemic lesions. 12 months after initiation of TCZ, clinical and sonographic findings showed no new disease activity, and VA FDG enhancement improved. MR-A showed regressive irregularities of both VA, whereas the black blood MR-A showed persistent contrast enhancement of the vessel walls.

Case 5:

A 62-year-old male presented in the Department of Internal Medicine with symptomatic recurrent hyponatremia and low back pain. FDG-PET, performed to rule out a paraneoplastic etiology of hyponatremia, showed no evidence of malignancy, but revealed active systemic vasculitis involving both VA. ESR (58 mm/h) and CRP (4.79 mg/dl) were elevated. High-dose MP, followed by oral prednisolone tapering, was administered. The etiology of hyponatremia was considered multifactorial (hypovolemia, adrenal insufficiency, most likely secondary to exogenous steroid administration, and/or

suspected SIADH in vasculitis). Cranial CT, performed for unclear confusion during follow-up, showed no cerebral ischemia. Cranial MRI for additional primary hypogonadism of most likely pituitary etiology showed subacute cerebellar ischemia. 8.5 months after the initial diagnosis of large vessel vasculitis, rituximab was initiated for suspected disease progression and was then administered at 6-month intervals. Nevertheless, recurrent anterior ischemic optic neuropathy (AION) resulted in deterioration of vision. Approximately 3.3 years after initial diagnosis of large vessel vasculitis, the patient presented in our department with repeated bilateral vertebrobasilar strokes with stenosis of the left VA and occlusion of the right VA, as demonstrated by MR-A and CDUS. Dabigatran for additional persistent atrial fibrillation and ASS were started, as well as high-dose MP therapy, followed by oral prednisolone tapering. B cells were still depleted (2/µl) after the last rituximab administration (4 months before). Temporal artery biopsy and another FDG-PET showed no signs of active vasculitis. However, both biopsy and FDG-PET were performed after multiple immunotherapies. Given the clear constellation of findings at initial diagnosis (headache, B-symptoms, recurrent AION, elevated ESR, and large-vessel vasculitis involving the temporal artery and VA in FDG-PET), subsequent vertebrobasilar ischemias and constrast-enhancing VA stenosis on MR-A, diagnosis of GCA was confirmed. Since treatment with rituximab was insufficient, immunotherapy was switched to TCZ. After 3 months, clinical and laboratory findings were unremarkable. At 6-month follow-up, VA stenoses remained unchanged, but MRI showed new ischemic vertebrobasilar stroke caused by persistent inflammatory disease activity. At 12-month follow-up, the patient was clinically stable. FDG-PET showed no signs of active vasculitis and MRI was without new ischemia. 14 months after initiation of TCZ therapy, the patient died of a septic shock due to a soft tissue infection of the leg. At that time, the patient was still on immunotherapy (prednisolone 5 mg/d, TCZ 162 mg s.c. weekly).

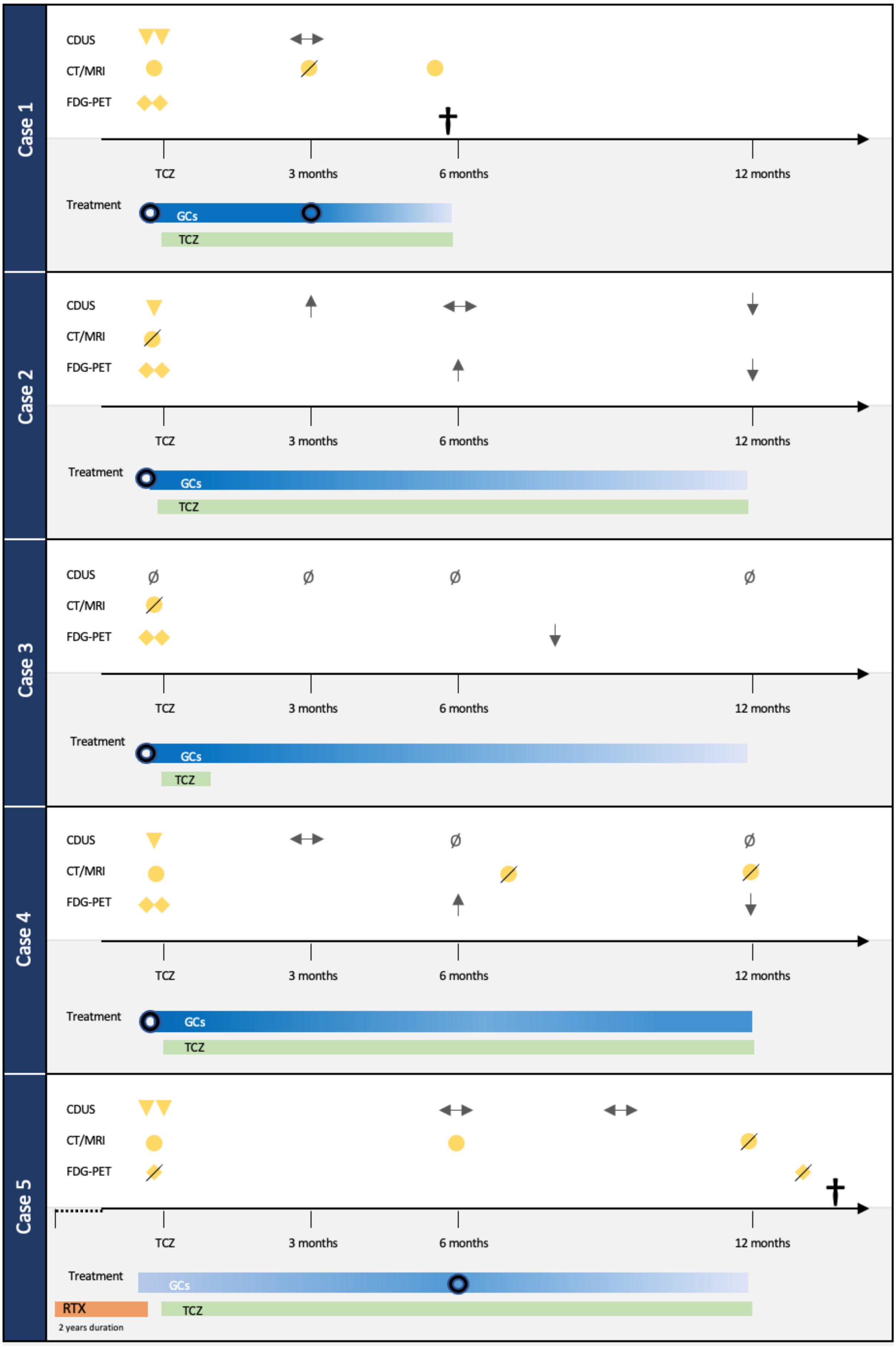

Colour Doppler ultrasound (CDUS): stenosis of 1 vertebral artery (VA), stenosis of 2 VA, ↑ increase of stenosis, ↓ decrease of stenosis, ↔ constant stenosis, Ø no stenosis. Magnetic resonance imaging (MRI): vertebrobasilar ischemia, no new vertebrobasilar ischemia. 18F-fluorodeoxyglucose (FDG) positron emission tomography (PET): FDG uptake of 1 VA, FDG uptake of 2 VA, no FDG uptake of VA, ↑ increase of FDG uptake, ↓ decrease of FDG uptake
Treatment: GCs, glucocorticosteroids; TCZ, Tocilizumab; RTX, Rituximab; high-dose intravenous methylprednisolone
† patient died

Figure S1: Course of TCZ patients. In cases 1-4, first diagnosis of GCA and initiation of TCZ therapy coincided, whereas in patient 5 initial diagnosis of GCA was made approx. 3.3 years before initiation of TCZ.
